# Expression of *BLIMP1/PRMT5 *and concurrent histone H2A/H4 arginine 3 dimethylation in fetal germ cells, CIS/IGCNU and germ cell tumors

**DOI:** 10.1186/1471-213X-8-106

**Published:** 2008-11-07

**Authors:** Dawid Eckert, Katharina Biermann, Daniel Nettersheim, Ad JM Gillis, Klaus Steger, Hans-Martin Jäck, Annette M Müller, Leendert HJ Looijenga, Hubert Schorle

**Affiliations:** 1 Department of Developmental Pathology, Institute of Pathology, University of Bonn, Germany; 2 Institute of Pathology, University of Bonn, Germany; 3 Department of Urology and Pediatric Urology, University of Giessen, Germany, Institute of Pathology, University of Giessen, Germany; 4 Division of Molecular Immunology, Nikolaus-Fiebiger-Zentrum, Universität Erlangen-Nürnberg, Erlangen, Germany; 5 Department of Pathology Erasmus MC-University Medical Center Rotterdam (Daniel den Hoed Cancer Center) Josephine Nefkens Institute, Rotterdam, the Netherlands

## Abstract

**Background:**

Most testicular germ cell tumors arise from intratubular germ cell neoplasia unclassified (IGCNU, also referred to as carcinoma *in situ*), which is thought to originate from a transformed primordial germ cell (PGC)/gonocyte, the fetal germ cell. Analyses of the molecular profile of IGCNU and seminoma show similarities to the expression profile of fetal germ cells/gonocytes. In murine PGCs, expression and interaction of Blimp1 and Prmt5 results in arginine 3 dimethylation of histone H2A and H4. This imposes epigenetic modifications leading to transcriptional repression in mouse PGCs enabling them to escape the somatic differentiation program during migration, while expressing markers of pluripotency.

**Results:**

In the present study, we show that BLIMP1 and PRMT5 were expressed and arginine dimethylation of histones H2A and H4 was detected in human male gonocytes at weeks 12–19 of gestation, indicating a role of this mechanism in human fetal germ cell development as well. Moreover, BLIMP1/PRMT5 and histone H2A and H4 arginine 3 dimethylation was present in IGCNU and most seminomas, while downregulated in embryonal carcinoma (EC) and other nonseminomatous tumors.

**Conclusion:**

These data reveal similarities in marker expression and histone modification between murine and human PGCs. Moreover, we speculate that the histone H2A and H4 arginine 3 dimethylation might be the mechanism by which IGCNU and seminoma maintain the undifferentiated state while loss of these histone modifications leads to somatic differentiation observed in nonseminomatous tumors.

## Background

In males aged 15 – 34 years, type II testicular germ cell tumors (TGCT), i.e. seminomas and nonseminomas, are the most common malignancies with fatal outcome [1] accounting for up to 60% of all malignancies in young man. The incidence of this type of cancer has been steadily increasing throughout the last decades [2]. The tumors arise from a neoplastic precursor, the carcinoma *in situ *(CIS)/*intratubular germ cell neoplasia unclassified *(IGCNU) and develop into seminoma and/or nonseminoma (including embryonal carcinoma, teratomas, yolk sac tumors and choriocarcinomas) [3]. The IGCNU lesions are believed to arise by delayed or blocked maturation of primordial germ cells (PGC)/gonocytes during early fetal development [4]. The recently identified markers for IGCNU and seminoma, namely the markers of pluripotency *OCT3/4 *and *NANOG *further support this model [5-10].

Expression of pluripotency genes is detected in embryonic stem cells (ES) and the inner cell mass of the early embryo. Additionally murine and human ES cells need to be cultured in the presence of factors inhibiting differentiation, although there are species specific differences [11,12]. In PGCs, early gonocytes and IGCNU as well as seminoma lesions some of these markers of pluripotency are expressed, although differences have been reported [13,14]. According to the current model, PGCs actively suppress somatic differentiation programs by epigenetic modifications, a mechanism which might also account for IGCNU and seminoma [15]. Recent data in mice demonstrate that suppression of somatic differentiation programs in PGCs is mediated by a complex of two proteins, Blimp1 (B-Lymphocyte induced maturation protein-1; PRDM1) and Prmt5 (protein arginine methyltransferase-5). Upon arrival in the genital ridge the PGCs differentiate to become gonocytes and the Blimp1/Prmt5 complex is translocated in the cytoplasm and subsequently, Blimp1 is downregulated. Targeted deletion of Blimp1 leads to loss of PGCs short after specification due to differentiation. The Blimp1-deficient PGCs display an insufficient repression of markers indicative for somatic differentiation such as *HoxB1 *[16]. Blimp1 is a transcriptional repressor harboring an N-terminal PR-SET domain, 5 zinc-finger domains and an acidic domain at the C-terminus. In murine PGCs the Blimp1/Prmt5 complex mediates symmetrical methylation of histones H2A and H4 at arginine 3 (H2AR3me2s, H4R3me2s), resulting in widespread epigenetic modification leading to transcriptional repression [17].

In the present study, we investigated the expression of BLIMP1/PRMT5 during human fetal germ cell development and in testicular germ cell tumors. Analyzing human fetal tissues, we found BLIMP1/PRMT5 colocalized in gonocytes at weeks 12 – 19 of pregnancy, supporting a role in human germ cell development. Furthermore BLIMP1/PRMT5 is expressed in IGCNU and seminoma, but downregulated in nonseminomatous GCTs. Since the nuclear localization of BLIMP1 correlated with the presence of the histone modifications H2AR3me2s and H4R3me2, our data help in explaining the undifferentiated/fetal state of IGCNU and seminoma.

## Results

### Normal germ cell development

**Figure 1:**
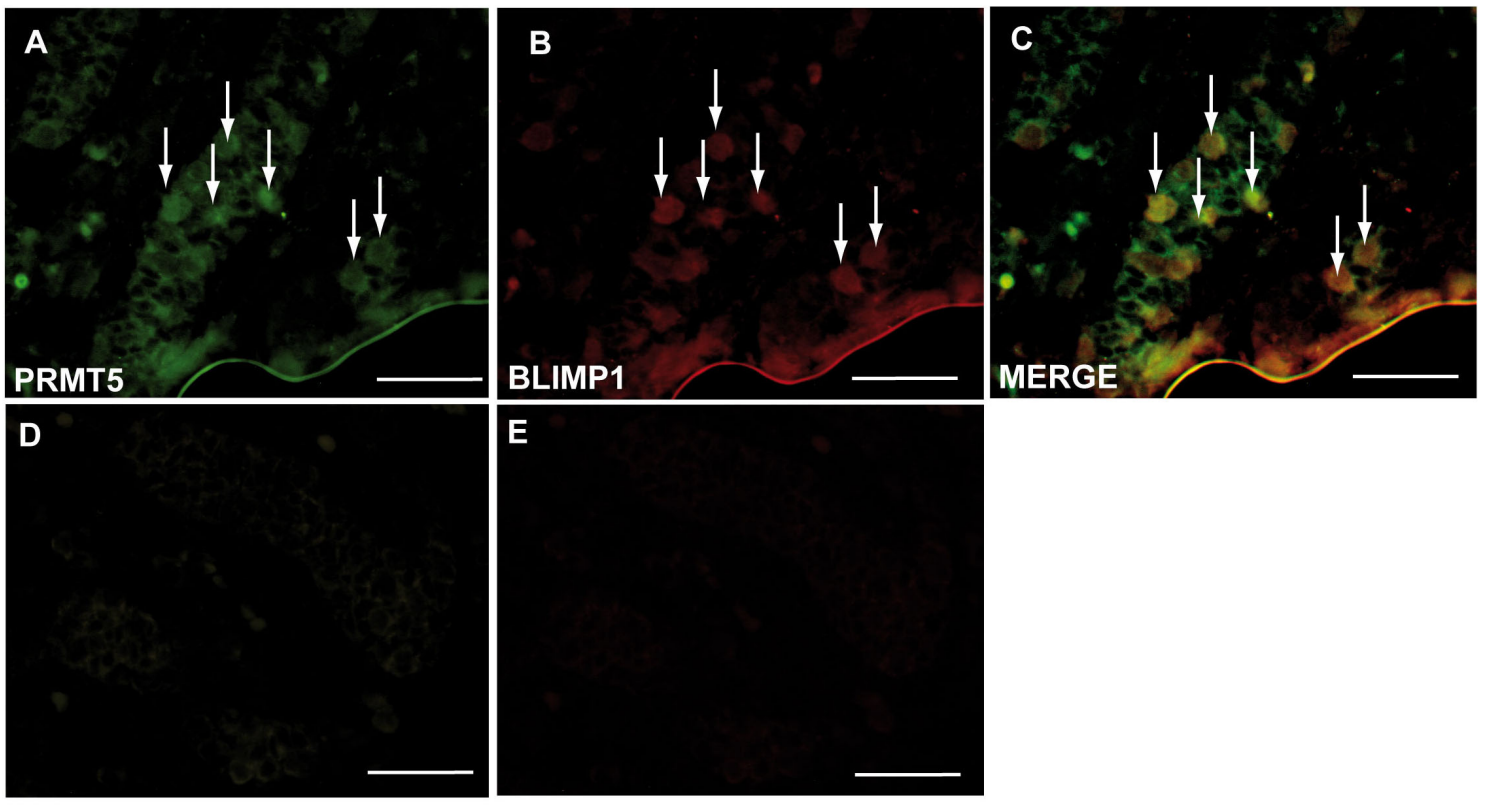
**Human fetal gonocytes at 12^th ^week of pregnancy**. Sections of human fetal gonocytes at 12^th ^week of pregnancy subjected to antibody staining towards BLIMP1 (A), PRMT5 (B) and overlay (C). D and E no primary antibody controls. Arrows indicate exemplary germ cells. Bar = 50 μm.

**Figure 2:**
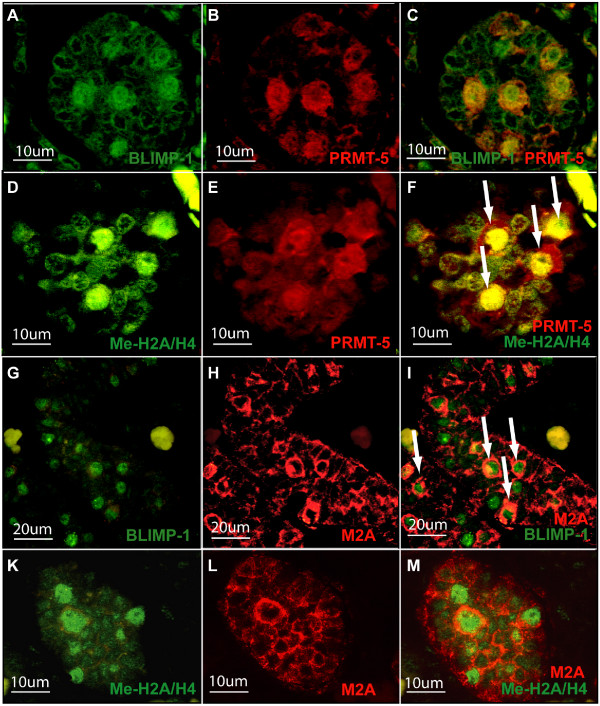
**Human fetal gonocytes at 19^th ^week of pregnancy**. Sections of human fetal gonocytes at 19^th ^week of pregnancy subjected to antibody staining towards (**A**) BLIMP1, (**B**) PRMT5, (**C**) Merge of BLIMP1 and PRMT5, (**D**) PRMT5, (**E**) methylated H2A/H4, (**F**) merge of PRMT5 and methylated H2A/H4 (**G**) BLIMP1 (**H**) M2A antigen, (**I**) merge of BLIMP1 and M2A, (**K**) methylated H2A/H4, (**L**) M2A, (**M**) merge of methylated H2A/H4 and M2A.

Data from murine embryos indicate, that the murine homologs of BLIMP1 and PRMT5, are expressed in PGCs from specification on up to their arrival in the genital ridge [16,17]. Short thereafter, these cells differentiate to become gonocytes and the Blimp1/Prmt5 complex is translocated in the cytoplasm and subsequently, Blimp1 is downregulated. In order to test whether human BLIMP1 and PRMT5 are detected in human fetal PGCs/gonocytes, immunohistochemical analyses were performed on human fetal material. On the 12^th ^week of pregnancy migrating gonocytes coexpressing PRMT5 and BLIMP1 were detected, (Fig. 1, compare A to B, merged in C, arrows). Next, testes from the 19^th ^week of pregnancy were analyzed. By this time gonocytes gradually differentiate into prespermatogonia and migrate towards the periphery of the emerging seminiferous tubules to settle down in their niche [18]. Both BLIMP1 (Fig. 2A) and PRMT5 (Fig. 2B) were detected at this stage in gonocytes. PRMT5, in contrast to BLIMP1, was detected both in the nucleus and in the cytoplasm. Since the murine Blimp1/Prmt5 complex has been described to mediate symmetrical dimethylation of arginine 3 on histone H2A and/or H4 tails (H2AR3me2s/H4R3me2s) [17] immunohistochemical analysis to detect this modification was performed (Fig. 2D). Co-staining of PRMT5 revealed that the cells displaying high nuclear levels of PRMT5 are in fact positive for the H2AR3me2s/H4R3me2s histone mark (Fig. 2E and 2F, merged). To further analyze the population of cells expressing BLIMP1 we performed double labeling experiments using BLIMP1 (Figure 2G) and the gonocytal markers M2A[19] (Figure 2H). BLIMP1/M2A double positive signals were detected in most gonocytes (Figure 2I, arrows). Double labeling for H2AR3me2s/H4R3me2s (Fig 2K) combined with M2A (Fig. 2L) showed, that the M2A positive gonocytes displayed H2AR3me2s/H4R3me2s modifications (Fig 2M). Again, these findings were in accordance with the situation in mice, where the Blimp1 protein is downregulated and the H2AR3me2s/H4R3me2s methylation is gradually lost when germ cells proceed to prespermatogonia [17].

**Figure 3:**
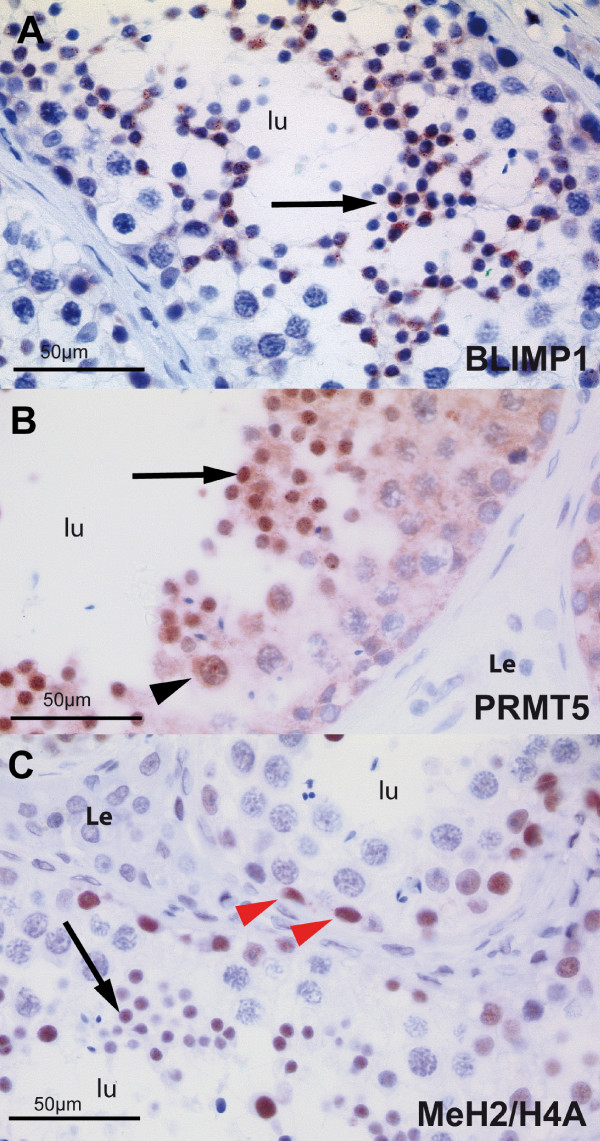
**Human adult testis**. Sections of normal human adult testis stained for BLIMP1 (**A**), PRMT5 (**B**) methylated and dimethylated histones H2A/H4 (**C**). **(A) **A seminiferous tubule is shown with normal spermatogenesis. Spermatogonia, spermatocytes, and Sertoli cells are devoid of the staining, while nuclear and cytoplasmatic staining occurs in round spermatids (large arrow). (**B**) Staining with PRMT5 antibody shows low expression of PRMT5 in the nuclei of spermatocytes (arrowhead), and strong nuclear staining in round spermatids (large arrow). (**C**) Positive staining with Me H2A/H4 occurs in spermatogonia (red arrowheads) and round spermatids (arrow), but not in spermatocytes. **lu **lumen of the seminiferous tubule; **Le **Leydig Cells.

Since the murine Blimp1/Prmt5 complex was specifically detected in early germ cells but not in prespermatogonia[16] we next asked whether PRMT5, BLIMP1 and dimethylated histone H2A and H4 could be detected in adult human testes. BLIMP1 was detected in the cytoplasm of round spermatids (Fig. 3A, arrow) and PRMT5 was found in the nuclei and in the cytoplasm of spermatocytes (Fig. 3B arrow) and round spermatids (Fig. 3B black arrowhead). H2AR3me2s/H4R3me2s modification is detected in type A spermatogonia (Fig. 3C red arrowhead), as well as round and elongated spermatids, (Fig. 3C arrow). The cytoplasmatic localization of BLIMP1 in adult testes excludes the functional interaction with PRMT5 and the resulting epigenetic modification. This implicates an alternative mechanism of H2AR3me2s/H4R3me2s modification in adult testes.

### Type II TGCTs

**Figure 4:**
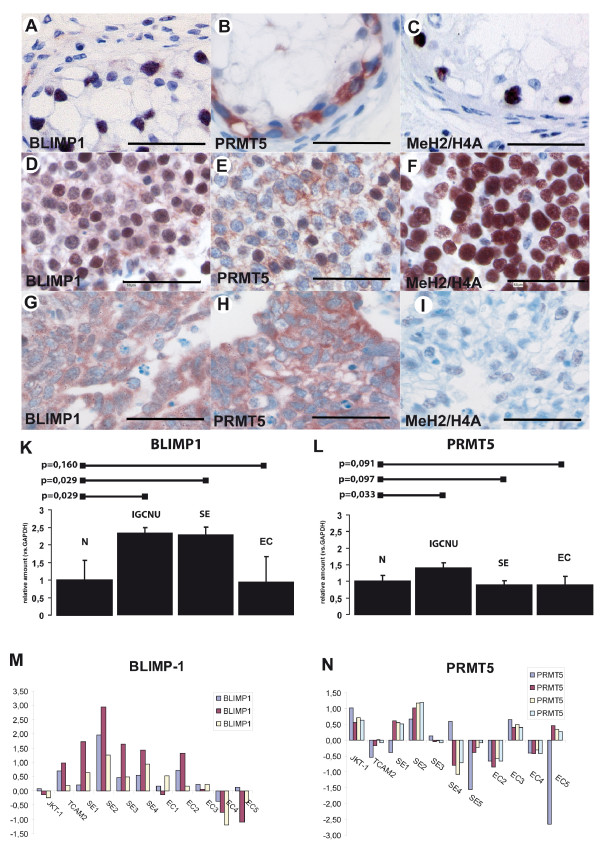
**Human germ cell tumors**. Sections of neoplastic germ cells of IGCNU (**A-C**), seminoma (**D-F**), embryonal carcinoma (**G-I**) stained for BLIMP1 (**A**, **D**, **G**), PRMT5 (**B**, **E**, and **H**) and methylated histones H2A/H4 (**C**, **F**, **I**). In Figure **A-C **tubules with IGCNU are shown with consistent nuclear expression of BLIMP1 and Me H2A/H4 in neoplastic germ cells (**A**, BLIMP1; **C**, Me H2A/H4). PRMT5 is expressed in the cytoplasm of neoplastic germ cells (**B**). Notice that no expression is present in Sertoli cells. In Figures **D-F **expression in seminomas is presented. Notice the variation of the expression of BLIMP1, being low or moderate in the majority of the cells (**D**). PRMT5 is expressed in the cytoplasm of most seminoma cells, but some neoplastic cells also show nuclear staining (**E**). Figure **F **shows a strong nuclear staining of MeH2A/H4 in most seminoma cells. Size bar is 50 μm. Quantification of the relative expression of BLIMP1 (**K**) and PRMT5 (**L**) normalized to β-Actin and compared to normal testicular tissue. Bars above the graph indicate p-values. (**M, N) **Expression values for BLIMP1 (**M**) and PRMT5 (**N**) from independent Affymetrix expression analyses (as referred in 23). Data are plotted as Log2 (y-axis) after normalization. Abbreviations: Normal testicular tissue (N), IGCNU, seminoma (SE), embryonal carcinoma (EC).

We next examined various TGCTs for the presence of BLIMP1/PRMT5 and H2AR3me2s/H4R3me2s. As shown in Figure 4, IGCNU show nuclear BLIMP1 staining (Fig. 4A), cytoplasmatic PRMT5 staining (Fig. 4B) and dimethylation of H2A/H4 (Fig. 4C). Seminomas show predominant nuclear BLIMP1 signal (Fig. 4D) sparse nuclear PRMT5 signal (Fig. 4E) as well as a strong and homogenous signal for H2AR3me2s/H4R3me2s (Fig 4F). In embryonal carcinoma, expression of BLIMP1 (Fig. 4G) and PRMT5 (Fig. 4H) was weak and cytoplasmatic. As expected, histone H2AR3me2s/H4R3me2s methylation (Fig. 4I) was barely detectable and heterogeneous. Yolk sac tumors teratomas and choriocarcinomas stained focally and cytoplasmatic for BLIMP1 and PRMT5 (not shown). Focal cytoplasmatic expression of BLIMP1 and PRMT5 was also observed in differentiated parts of teratoma, while chorioncarcinomas were negative for both proteins. A summary of the results of the immunohistochemical studies is given in Table 1.

**Table 1:** Expression of BLIMP1, PRMT5 and dimethylated histone H4/H2A in normal and neoplastic testicular tissues

	BLIMP1	PRMT5	H4R3me2s/H2Ame2s
Normal fetal testis			
Gonocytes	+++ (n)	+++ (n,c)	+++ (n)
Pre-spermatogonia	-	-	
Normal adult testis (N = 18)			
Spermatogonia	-	-	++ (n)
Pachytene spermatocytes	-	+ (n)	-
Round spermatids	+ (n,c)	++ (n)	++ (n)
Elongated spermatids	-	-	-
Testicular germ cell tumors			
IGCNU (N = 15)	+++ (n) 85–100%	+++ (c) 75–95%	+++ (n) 90–100%
Seminoma (N = 20)	++ (n) 10–75%	++ (n+c) 30–85%	++ (n) 20–80%
Embryonal carcinoma (N = 15)	+/-(c)* 15–80%	++ (c) 15–80%	(+)*
Teratoma (N = 5)	+ (n, c)	+ (n, c)	+ (n)
Chorioncarcinoma (N = 3)	+ (c)	-	-

N, number of cases; n, nuclear staining; c, cytoplasmatic staining

+ weak; ++ moderate; +++, strong expression; -, no expression detectable

*, only single tumor cells were detected as positive.

In order to quantify the expression of BLIMP1 and PRMT5 we performed RT-PCR analyses on normal testicular tissue as well as on various TGCTs. The RNA levels measured were first normalized to βActin and then calculated as relative expression with normal testicular tissue (N) set at 1. Expression of BLIMP1 was significantly higher in IGCNU (p = 0.029) containing testicular parenchyma and seminoma (Fig. 4K), but not in embryonal carcinoma (EC) (p = 0.16), which was comparable to normal testicular tissue. In contrast, PRMT5 was moderately higher in IGCNU (p = 0.033), while embryonal carcinoma (p = 0,091) and seminoma (p = 0,091) express a similar level of PRMT5 compared to normal testicular tissue (Fig. 4L). These data could be confirmed, using a whole genome expression DNA-Array as reported before [20]. Here, the same pattern was observed (see Fig. 4M and 4N).

**Figure 5:**
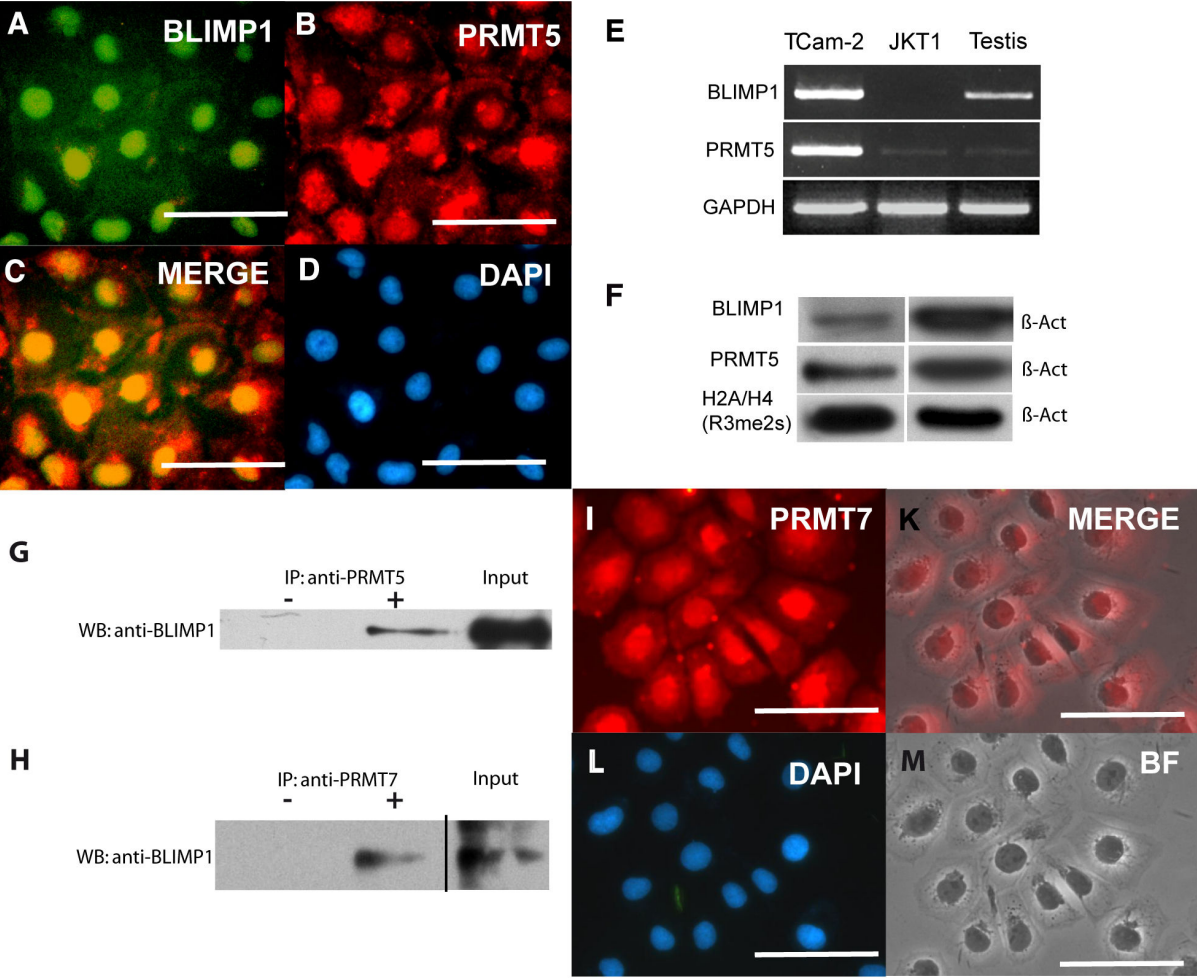
**Analysis of TCam-2 seminoma cell line**. (**A**-**B**) Immunohistochemistry using the antibodies indicated. (**C**) Merge of (**A**) and (**B**). (**D**) Counterstaining with DAPI to detect nuclei. (**E**) RT-PCR cell lines TCam2 and JKT1 as well as Testis detecting expression of the indicated genes. (**F**) Western Blot of protein lysate from TCam2 cells detecting the proteins indicated. (**G**) Co-IP experiment using antibody to PRMT5 for IP and antibody to BLIMP-1 to detect potential interaction. – no Antibodyl; + IP using PRMT5 Antibody; Input Control. (**H**) Co-IP experiment using antibody to PRMT7 for IP and antibody to BLIMP-1 to detect potential interaction. – no Antibodyl; + IP using PRMT5 Antibody; Input Control. (I-M) Immunohistochemistry using the PRMT7 antibody (I), (**K**) Merge of (**I**) and (**M**), (**L**) Counterstaining with DAPI to detect nuclei, (M) brightfield image. Scale Bar indicates 25 μm.

Finally, we asked whether BLIMP1/PRMT5 and modification of histone H2A and H4 could be detected in TCam-2, a cell line derived from a seminoma patient [21,22]. Here, we were able to detect BLIMP1 in the nucleus, PRMT5 in the nucleus and the cytoplasm (Fig. 5A–C). RT-PCR analyses showed that BLIMP1 and PRMT5 are expressed in TCam-2 cells (Fig. 5E) and absent JKT1 cells, in agreement with Affymetrix data (Fig. 4M and 4N). Of note, the findings on the JKT-1 cell line are in concordance with the conclusion that it is not a seminoma cell line [22,23]. Western blot analysis confirmed these results, showing that BLIMP1 and PRMT5, as well as the modified Histones H2A and H4 (Fig. 5F) can be detected. Next, we performed a CoIP on extracts from TCam-2 cells and were able to detect a signal for Blimp1 in material immunoprecipitated with PRMT5 antibody (Fig. 5G). This result demonstrates for the first time that PRMT-5 and BLIMP-1 interact biochemically.

We had shown, that nuclear BLIMP1 and methylated H2A and H4 are expressed in IGCNU and seminoma, yet these cells express either little or cytoplasmic PRMT5 (Fig. 4A–F). We speculated that another methytransferase cooperating with BLIMP1 might be able to compensate PRMT5 function and help in establishing this methylation pattern. PRMT7 which is like PRMT5 a type II methyltransferase seemed a potential candidate since both PRMT5 and PRMT7 have been demonstrated to mediate symmetric arginine dimethylation of sm Proteins required for the spliceosome [24]. The CoIP experiment (Fig. 4H), demonstrates that BLIMP1 and PRMT7 interact biochemically. In addition PRMT7 shows a strong nuclear signal in TCam-2 cells (Fig. 4I–M). These results indicate that in germ cell tumors, both PRMT5 and PRMT7 might cooperate with BLIMP1 to establish dimethylation of H2A and H4.

## Discussion

In this study, we analyzed the expression of the putative inhibitor complex of germ cell differentiation BLIMP1 and PRMT5 on mRNA and protein level and the presence of the resulting repressive histone modifications H2A/H4R3me2s in human fetal and adult germ cells as well as TGCTs. We found BLIMP1 and PRMT5 localized in the nuclei of gonocytes, and the latter also in the cytoplasm, and could show the presence of the resulting dimethylation of H2A/H4 at arginine 3. In IGCNU a strong nuclear signal of BLIMP1 and of H2K3me2s/H4K3me2s was detected, whereas PRMT5 signal was cytoplasmatic in IGCNU and heterogeneous in seminomas.

The expression in fetal gonocytes in humans described here is in concordance to the observations made in mouse [17] indicating a conserved role of the nuclear localized BLIMP1/PRMT5 complex between mouse and man. Recently the transcriptional repressor BLIMP1 has been shown to be a crucial determinant of the germ cell lineage in mice [16]. This Krüppel-type zinc-finger containing protein interacts with the arginine methyl-transferase PRMT5 resulting in a symmetrical methylation at arginine 3 of histone H4 and H2A (H4R3me2s/H2Ame2s). The methylation in turn represses transcription[17] and therefore might be important for suppressing the somatic cell fate and keeping germ cells in a pluripotent state. In fact, in mice Blimp1-deficent germ cells show inconsistent repression of *HoxB1*, a hallmark of germ cell specification and fail to express *Stella *a marker of undifferentiated germ cells [16]. Also, recent studies showed, that abrogation of the *Drosophila melanogaster *homolog of PRMT5, *Capsuleen/dart5*, is essential for germ cell specification and maintenance [25,26]. Interestingly, Blimp1 expression is lost in PGCs which are cultured in the presence of basic FGF and LIF [15] and gradually become embryonic germ cells [27-29]. Hence the BLIMP1/PRMT5 interaction resulting in H2A/H4 modification might lead to repression of premature differentiation during human fetal germ cell development. As a consequence prolonged expression of BLIMP1/PRMT5 could result in persistence of undifferentiated gonocytes into adulthood.

It is believed that those persisting gonocytes give rise to IGCNU the common precursor lesion of all type II TGCTs [4]. Indeed, we detected BLIMP1 protein and the characteristic modification of histones H2A and H4 not only in gonocytes but also in IGCNU and in seminoma supporting a PGC/gonocyte origin of IGCNU and therefore GCT [5,8,30,31]. PRMT5 however, is not detectable in nuclei of IGCNU, and displays only a sparse nuclear localization in seminoma cells. We found that another type II protein arginine methyltransferase, PRMT7 is expressed in TCAM2 seminoma cells and that PRMT7 interacts with BLIMP1 as well. So we speculate that in IGCNU and seminoma, BLIMP1 recruits PRMT7 to compensate for the lack of nuclear PRMT5 to mediate H2A and H4 dimethylation.

Upon progression of IGCNU to nonseminomas signal intensity of BLIMP1 decreased and subcellular localization changed. As a consequence, H2A/H4 modification decreased and became heterogeneous in nonseminomas. Hence, the loss of the repressive histone modifications allows further uncontrolled differentiation observed in nonseminomas.

## Conclusion

Taken together we propose the following model for development of germ cell neoplasia. First, coexpression and nuclear localization of the BLIMP1/PRMT5 complex leads to histone H2A/H4 dimethylation which results in transcriptional silencing of genes responsible for somatic differentiation in PGCs. Upon differentiation to prespermatogonia, this complex is downregulated and the H2A/H4 marks are lost. Aberrant constitutive histone H2A/H4 arginine 3 dimethylation allows the cells to escape the regular differentiation program resulting in their persistence into adulthood. These cells eventually progress into IGCNU, displaying the H2A/H4R3me2s modification as well. Since the subcellular localization of PRMT5 excludes PRMT5-dependent histone H2A/H4 modification in IGCNU we propose that BLIMP1 might act in cooperation with PRMT7. This mechanism persists in seminoma where the H2A/H4R3me2s modifications can be observed which explains the undifferentiated nature of the tumor cells. Translocation of BLIMP1 into the cytoplasm leads to breakdown of histone H2A/H4 dimethylation and subsequently to the activation of the differentiation programs and therefore the conversion from IGCNU into a nonseminomatous germ cell tumors.

## Methods

### Sample Handling and Characterization

Formalin fixed, paraffin embedded testicular tissues from 46 patients with GCTs (20 seminomas, 15 embryonic carcinomas, 5 Teratomas, 3 yolk sac tumors and 3 choriocarcinomas were collected for this study from archives of Departments of Pathology of University Medical Centers Bonn. Adjacent testicular parenchyma containing IGCNU were studied in 15 cases[32]. All tumors were classified according to the WHO classification of tumors based on their histology by two independent pathologists. Fresh frozen samples of each of normal testicular tissues (n = 3), seminoma (n = 3), mixed germ cell tumors (n = 3), IGCNU (n = 5) and embryonal carcinomas (EC) (n = 3), as well as RNA extracts of TCam2 [33] and JKT-1[34] cell lines, of which TCam2 resembles a seminoma-like cell-line [21-23], were additionally available for this study. Use of the tissue for scientific purposes was approved by the Institutional Regional Committee for Ethics.

### RT-PCR and quantitative image analysis

Total RNA from at least three samples per tumor entity was extracted with TRIzol (Invitrogen, Karlsruhe, Germany) according to manufacturer's instruction. cDNA-syntesis was performed using SuperScript III reverse transcriptase (Invitrogen, Karlsruhe, Germany) and Oligo d(T)_12–18_(Invitrogen, Karlsruhe, Germany) and 100 ng of total RNA according to manufacturers instructions. PCRs were carried out in triplicates with following Primers: *BLIMP1 *F: 5'-GGGTGCAGCCTTTATGAGTC-3'; *BLIMP1 *R: 5'-CCTTGTTCA TGCCCTGAGAT-3'; *PRMT5 *F: 5'TTGCCGGC TACTTTGAGACT-3'; *PRMT5 *R: 5'-AAGGCAGGA AAGCAGATTGA-3'; *GAPDH*-F: 5'-TGGTATCGTGGAA GGACTCATG AC-3; *GAPDH R*: 5'-ATGCC AGTGAGCTTCCCGTTCAGC-3'. (β-Act: 25 cycles BLIMP1 and PRMT5: 30 cycles). After agarose gel electrophoresis of the PCR-products band intensity was measured after RT-PCR with the image analysis software ImageJ 1.37 v (National Institutes of Health, USA, ) in triplicates and normalized to the according GAPDH band.

### Co-Immunoprecipitation

Co-IP was performed with DYNABEADS^® ^(Invitrogen, Carlsbad, USA) following manufacturers instructions. Immunopreciptation was performed with 1,5 μg anti-PRMT5 antibody (Chemicon, Temecula, USA) or anti-PRMT7 (Abcam, Cambrigde UK, 1:250). Western Blot with anti-BLIMP1 antibody followed (provided by H. M. Jäck).

### Western Blot

For protein analysis Mini-PROTEAN Electrophoresis Cell and Mini Trans-Blot system was used (BioRad, Munich, Germany). Proteins were isolated using RIPA-buffer and prepared using standard protocol and finaly electrophoresed at 30 mA for 90 min. The gel was blotted onto a PVDF membrane in a BioRad blotting chamber overnight at 30 V at 4°C according to published protocols. After blocking in PBSTM (PBS, 0.1% v/v Tween 20, 5% low fat milk powder) primary antibodies (anti-BLIMP1 1:400 (kind gift from H. Jäck), anti-PRMT5 1:200, Chemicon International, USA) were incubated in PBSTM for 3 h at RT. The secondary antibodies (anti-rabbit-HRP, anti-mouse-HRP: DAKO, Hamburg, Germany) were diluted 1:2000. Finally the membrane was incubated in 2 ml PierceSuper Signal West Pico chemiluminescent substrate (Perbio, Bonn, Germany) and the signal was detected using Kodak X-Ray film (Kodak, Stuttgart, Germany).

### Array Analysis

DNA Array Dataset used to analyze BLIMP1/PRMT5 expression in Seminoma, embryonal carcinoma, TCam2 and JKT1 were generated as described [32].

### Immunohistochemistry

For immunohistochemistry on paraffin-embedded tissue, dewaxed, 4-μm thick tissue sections were microwave-pretreated in citrate-buffer. Primary antibodies to PRMT5 (Upstate, Charlottesville, VA, 1:500), PRMT7 (Abcam, Cambrigde UK, 1:250) BLIMP1 (provided by H-M. Jäck, University of Erlangen, Germany 1:500) and H2AR3me2s/H4R3me2s (Abcam, Cambridge, UK, 1:2000) were used for detection. Immunohistochemistry was performed using the DAKO EnVision-AEC Kit and manufacturers protocol (DAKO, Hamburg, Germany) as previously described [7]. Briefly, endogenous peroxidase was blocked for 5 min in 0.03% H_2_O_2 _(diluted in distilled water). Sections were washed in Tris-buffered saline (TBS; 0.05 M Tris and 0.85% NaCl, pH 7.6) and incubated with primary antibodies overnight at 4°C. Thereafter, a HRP-labeled polymer conjugated with a secondary antibody was applied (DAKO EnVision-AEC KIT). Pictures were taken using a Leica microscope fitted with a JVC digital camera (Leica, Bensheim, Germany). Figures were assembled using Adobe CS3 software package. Merge of pictures was performed using ImageJ (NIH, US).

## Authors' contributions

DE, KB, DN, AG performed experiments, AG, HMJ, AMM and LL contributed material, KS helped in analysis of the Immunohistochemistry. DE, KB and HS conceived the experiments. DE, KB, LL and HS wrote the manuscript. All authors have seen and approved the final version.
